# Supplementary material for: A Global Screen for Assembly State Changes of the Mitotic Proteome by SEC-SWATH-MS
Source: Cell Syst. 2020 Feb 26;10(2):133–155.e6. doi: 10.1016/j.cels.2020.01.001 (PMC7042714; doi:10.1016/j.cels.2020.01.001)

Q9HCK8 | CHD8_HUMAN | CHD8 HELSNF1 KIAA1564
Monomer MW [kDa]: 290.519 Monomer expected elution fraction: 31

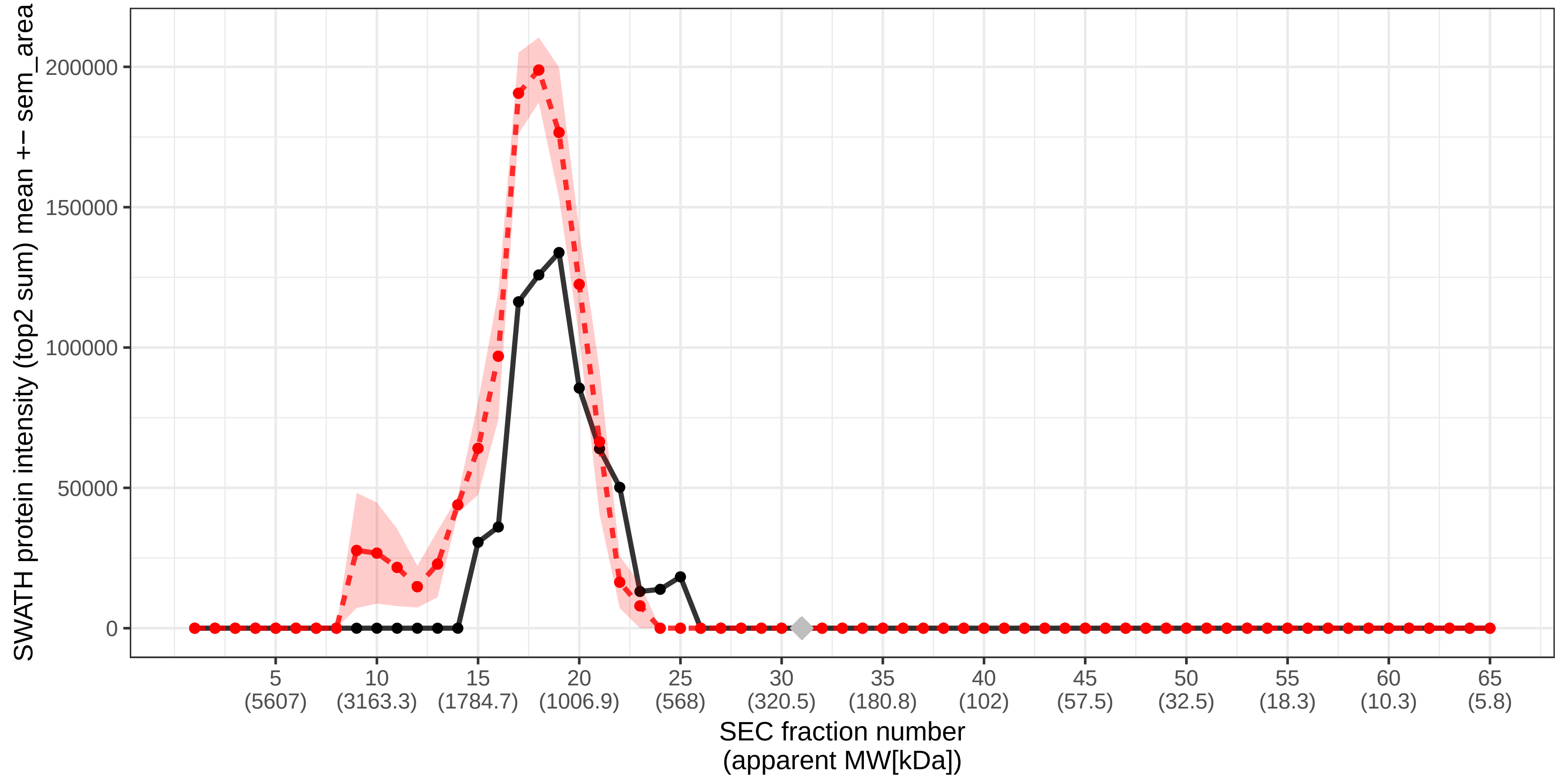

Supplement: Data S1. SEC-SWATH-MS Protein Chromatograms, Related to Figure 1 [file mmc6.zip › SECchrom_Q9HCK8_CHD8_HUMAN_CHD8_HELSNF1_KIAA1564.pdf]
